# Publication rate of scientific papers presented at the largest event on breast cancer research in Latin America

**DOI:** 10.3332/ecancer.2021.1259

**Published:** 2021-07-01

**Authors:** Sergio Nascimento, Rosemar Macedo Sousa Rahal, Leonardo Ribeiro Soares, Herik Jansen de Souza Pimentel, Tágara Oliveira Kamimura, Ruffo Freitas-Junior

**Affiliations:** 1 Faculty of Medicine, School of Medicine, The Federal University of Goiás, Rua 235 s/nº, Setor Leste Universitário, 74605050 Goiânia, GO, Brazil; 2 Advanced Center for Breast Diagnosis (CORA), Teaching Hospital, Federal University of Goiás, Primeira avenida s/nº, Setor Leste Universitário, 74605020 Goiânia, GO, Brazil; 3 School of Medicine, Alfredo Nasser University, Av Bela Vista, nº 26, Jardim Esmeraldas, 74905020 Aparecida de Goiânia, GO, Brazil; a https://orcid.org/0000-0003-1294-2524; b https://orcid.org/0000-0003-3619-0603; c https://orcid.org/0000-0002-9448-6114; d https://orcid.org/0000-0002-4411-0397; e https://orcid.org/0000-0002-5053-1040; f https://orcid.org/0000-0003-4145-8598

**Keywords:** breast neoplasms, bibliometrics, research report, journal article

## Abstract

**Purpose:**

Medical congresses allow scientific production to be appropriately disseminated and discussed. However, most of the scientific papers presented at medical congresses do not go on to be published in indexed journals. The present study aimed to determine the publication rate of papers presented at the Brazilian Breast Cancer Symposium (BBCS) and trends associated with publication over that timeframe.

**Methods:**

This was a retrospective, observational study evaluating scientific papers presented at the BBCS between 2012 and 2017. All the abstracts presented at the event within this timeframe were recorded. Next, a search for papers was made using online databases (BIREME/LILACS and MEDLINE/PubMed) and in the curricula of the authors on the Lattes Platform.

**Results:**

Overall, 543 abstracts of papers presented at the BBCS between 2012 and 2017 were included. Of these, 112 (20.6%) had been published in an indexed journal, mostly in English (67.0%), in journals with an impact factor of 2.0–3.0 (21.4%) and ≥1 year after presentation at the event (75.9%). The factors associated with publication were: study conducted in a public institution (*p* = 0.01), oral or commented poster presentation (*p* > 0.001) and study concerning rehabilitation following breast cancer (*p* = 0.04). There was a downward trend in the rate of publication of articles over the years (*p* = 0.01). Conversely, the impact factor of the publications increased significantly between 2012 and 2017 (*p* = 0.04).

**Conclusion:**

The publication rate of papers presented at the BBCS is low and remains consistent over the study period despite academic incentives and substantial awards.

## Introduction

Disseminating knowledge gained from scientific research is the first step for healthcare advancement. Safe and reliable sources of information are of vital importance, and priority must be given to scientific studies conducted with appropriate methodology [1–3]. In recent years, scientific development in the field of breast cancer has contributed to progress in the diagnosis of the disease, to consolidating new treatment strategies and to achieving more favourable oncologic outcomes [3–5].

Medical congresses allow scientific production to be appropriately disseminated and discussed. The authors of each study may present their results and discuss the study limitations as well as the practical implications of their results. Among other benefits, medical congresses promote continued education, the discussion of clinical cases, the formulation of institutional protocols and the launching of specialised bibliographic material, as well as allowing contact between individuals from different geographic regions [6]. In this respect, bibliometric studies within any given medical speciality can contribute by providing indicators related to the production and dissemination of scientific knowledge [1, 7]. These studies also allow the progress achieved in any given subject or topic within each area of interest to be evaluated [1, 2, 6].

Data published in Brazil show that most of the scientific papers presented at medical congresses do not go on to be published in indexed journals [8, 9]. At the Brazilian Congresses of Angiology and Vascular Surgery held between 2001 and 2003, the mean publication rate was 6.3% [9]. Of the papers presented at the Brazilian Congress of Mastology held in 2017, the publication rate was 5.4% [3]. However, there are no data available on the temporal evolution of the publication rate for the same congress. To the best of the authors’ knowledge, this is the first temporal study of scientific events in the field of breast cancer in Brazil.

The Brazilian Breast Cancer Symposium (BBCS) is an annual scientific event that focuses on research in breast cancer and related subjects. The venue is Goiânia, Goiás, in the country’s mid-western region. The event was created in 2010 and since then around 5,000 participants have been engaged, approximately 600 scientific papers have been presented, and approximately US$ 45,000.00 in awards has been distributed to researchers.

The objective of the present study was to evaluate the publication rate of papers presented at the BBCS between 2012 and 2017, as well as trends in the publication of these papers throughout this timeframe.

## Methods

This was a retrospective, observational study, with the unit of observation being the scientific papers presented at the BBCS between 2012 and 2017. This symposium was selected because it is the largest event on breast cancer research in Brazil and Latin America. The 2012–2017 period was selected to allow an interval of 3 years after presentation, since this period is considered an appropriate time for the publication of abstracts presented at a congress [1]. In 2011, the BBCS event was not held because the city of Goiânia was the venue for the Brazilian Congress of Mastology.

### Variables

The following data were collected: the title of the paper, its authors and the institution and state in which the study was conducted. The abstracts were classified according to type of presentation as: posters, commented poster presentations or oral presentations. The main topic of the study was classified into the following categories: ‘epidemiology’, ‘histology’, ‘radiology’, ‘systemic treatment’, ‘surgery’, ‘radiotherapy’, ‘rehabilitation’, ‘basic science’ and ‘other topics’. The institutions at which the studies were conducted were classified as: ‘public’, ‘private’ or ‘public-private partnership’.

To determine whether the study had been published, a search was initially made of the authors’ curricula, which are available on the Brazilian National Council for Scientific and Technological Development (CNPq)’s Lattes Platform (www.lattes.cnpq.br). Two investigators conducted the search independently, using the name of the first and last author of the paper presented at the congress. The last date for search must be given as 10 December 2020.

Next, a search was made for the papers on the online databases: BIREME/LILACS virtual health library (http://lilacs.bvsalud.org/) and PubMed (US National Library of Medicine, National Institutes of Health) (https://www.ncbi.nlm.nih.gov/pubmed). Finally, if the search using the authors’ names was unsuccessful, another search was made in the same databases using the title of the paper.

For the papers published in journals, agreement was evaluated between the article in the journal and the paper presented at the medical congress. Changes in the titles, authors, objectives, materials and methods, results and conclusions were evaluated.

The absolute number of publications was evaluated, together with the language in which the paper was published and the year and journal in which it was published (national or international), the type of study and the quality of the scientific evidence. The publication rate was obtained for each congress based on the ratio between the total number of papers published and the total number of papers presented at the congress.

The classification proposed by the Brazilian Medical Association (http://www/amb/org/br) was used to classify the degree of scientific evidence: (A) The most consistent experimental or observational studies (meta-analysis or randomised clinical trials); (B) Less consistent experimental or observational studies (other non-randomised clinical trials or observational studies or case–control studies); (C) Reports or case series (non-controlled studies) and (D) Opinion lacking critical evaluation, based on consensuses, physiological studies or animal models.

### Statistical analysis

The data collected were initially entered on an Excel spreadsheet (Microsoft Corporation, Redmond, USA; version 2013), and then analysed using the SPSS statistical software program (IBM Corporation, Armonk, USA; version 23.0). The data were characterised using absolute (*n*) and relative (%) frequencies. Nonparametric statistical tests and techniques were used in this study as required, with the normality of distribution being defined according to the Kolmogorov–Smirnov test. The exploratory analyses of the categorical variables were performed using multiple contingency tables, with Pearson’s chi-square test being applied, followed by post-hoc analysis [10]. Comparison between the journal’s impact factor and the type of institution was performed using the Mann–Whitney test. The trend in the impact factor between 2012 and 2017 was examined using Spearman’s correlation coefficient. Poisson regression was used based on the Annual Percentage Change (APC) of publications between the periods from 2012 to 2017. Significance level was defined as *p* < 0.05 for the entire statistical analysis.

### Ethical aspects

The data used are publicly available at the headquarters of the event’s organising committee (https://2020.bbcs.org.br/). For this type of study, formal consent is not required. All recommendations of good clinical practice were followed according to Brazilian law and the Helsinki Convention.

## Results

A total of 543 abstracts of scientific papers presented at the BBCS between 2012 and 2017 were included in the study. The majority of these papers refer to work conducted in public institutions (71.1%), originating in the state of Goiás (47.3%) and being presented in poster form (72.0%) (Table 1).

**Figure 1.**
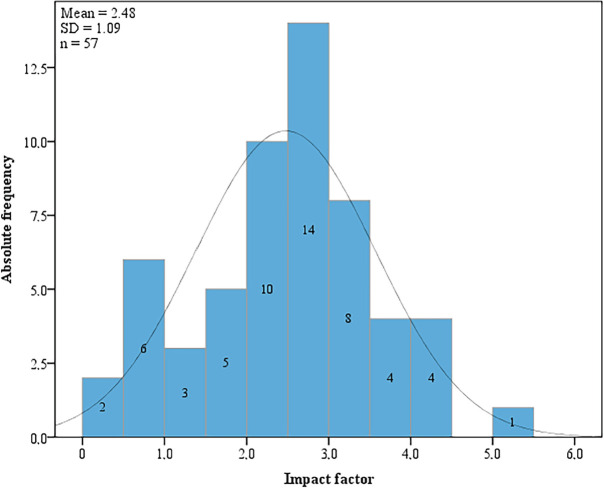
Histogram showing the distribution of the impact factor of the journals in which the papers were published.

Of all the abstracts presented, 112 (20.6%) were published in an indexed journal. Fifty-five (49.1%) articles were published in journals with no impact factor. The others papers were published in journals with an impact factor between 0.1 and 1.9 (14.3%), between 2.0 and 3.0 (21.4%) and greater than 3.0 (15.2%) (Figure 1). In most cases, the work was published a year or more after presentation at the event (75.9%) and published in English (67.0%) (Table 2).

**Figure 2.**
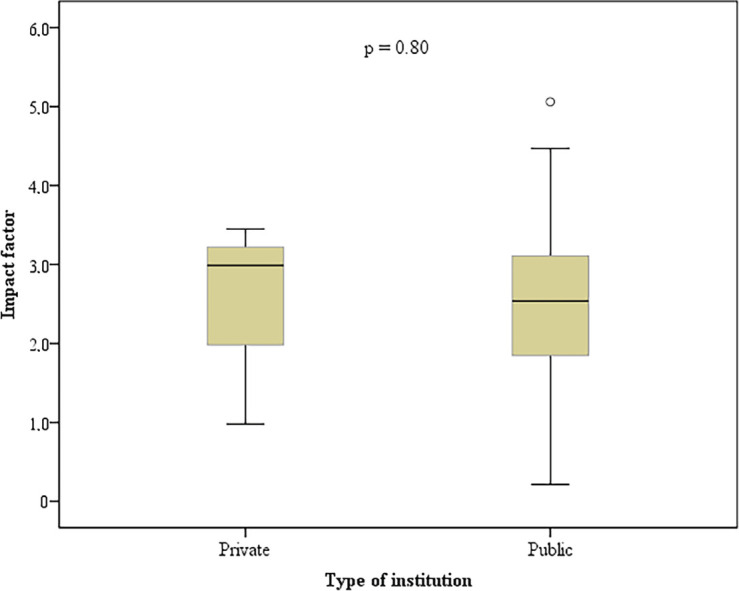
Boxplot comparing the impact factor with the type of institution in which the study was conducted.

The factors associated with publication of the scientific papers were: study conducted in a public institution (*p* = 0.01), oral or commented poster presentation (*p* < 0.01) and study concerning rehabilitation following breast cancer (*p* = 0.04) (Table 1). There was no statistically significant association between the journal’s impact factor and the type of institution in which the study was conducted (*p* = 0.80) (Figure 2). No statistically significant association was found between time to publication of the paper, language of publication or the degree of scientific evidence (Table 3). Table 4 shows the names of all the journals in which the publication rate of these papers was over 1%.

**Figure 3.**
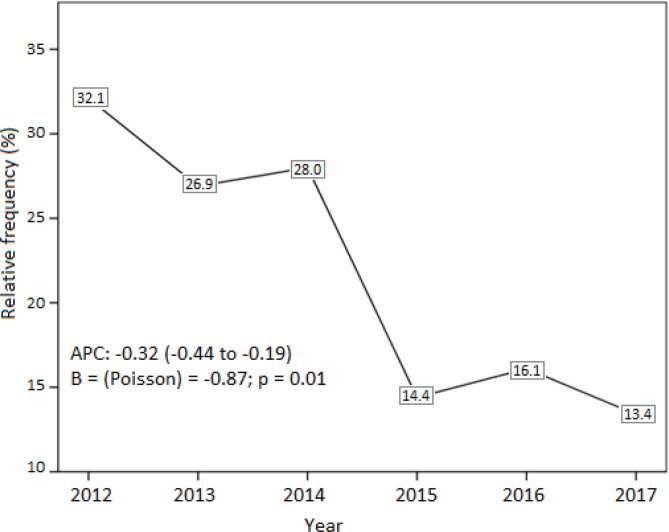
Line graph showing the publication rate in the periods from 2012 to 2017 and the result of the Poisson regression used from the APC.

**Figure 4.**
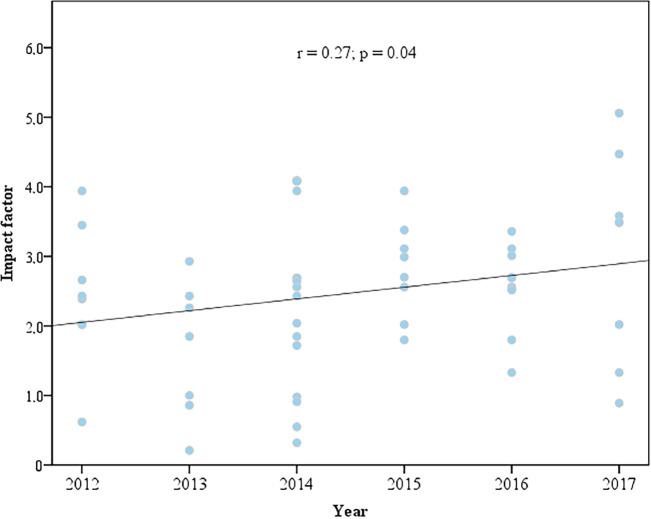
Scatter plot for the correlation between the journal’s impact factor and the year evaluated between 2012 and 2017 (Spearman’s correlation coefficient).

There was a downward trend in the rate of publication of articles over the years (*p* = 0.01) (Figure 3). In 2012, 17 of the 53 abstracts presented at the congress (32.1%) were published in an indexed journal, while, in 2017, 13 of the 97 papers presented were published (13.4%). Conversely, there was a significant increase in the impact factor of the publications between 2012 and 2017 (*p* = 0.04) (Figure 4). The distribution of the other characteristics according to the time evolution is shown in Table 5.

## Discussion

The dissemination of knowledge gained from scientific research represents a crucial step in the process of improving healthcare. As a prerequisite, care must be taken to ensure that the data in question originates from safe, reliable sources that prioritise scientific papers with appropriate methodology. The present study evaluated the publication rate of papers presented at the largest event on breast cancer research in Latin America. Papers for presentation at that event are previously selected by a committee specifically formed for this purpose and composed of professionals of renowned scientific expertise. Nonetheless, the average rate of publication was as low as 20.6% (ranging from 13.4% to 32.1%).

A possible factor associated with this low rate of publication that merits particular emphasis refers to the technical and methodological limitations of the studies conducted and presented at scientific events. In a study that analysed the quality of the abstracts presented at the congress of the Brazilian Society of Infectious Diseases, results showed that in around half the papers analysed the methodology was inadequately or incompletely described [11]. Among the published abstracts from the Brazilian Congress of Plastic Surgery, 88.5% had no statistical analysis, 76% had no impact factor and 52% had no citations [8]. In the present study, 55 (49.1%) abstracts were published in journals with no impact factor and another 16 (14.3%) with an impact factor between 0.1 and 2.0. Although this information does not necessarily imply poor scientific quality, it could reflect limitations in the methodology that resulted in the papers being published in journals with a lower impact factor.

In Brazil, the current model of scientific production is focused on postgraduate programmes and is associated with government incentives, when available [12, 13]. In this model, the publication process becomes dependent on personal and motivational factors present in the respective students and faculty, who not infrequently admit defeat after their paper is refused by the first journal to which it is submitted. In the University of São Paulo, one of the most renowned universities in Latin America, less than 50% of doctoral theses presented between 1990 and 2000 were published within a 5-year period [7]. Other factors such as financial limitations, lack of institutional incentives and a lack of technical support may also discourage scientific publication of a recently concluded study [14, 15].

We observed in the analysis using Poisson regression that the publication rate has been decreasing over the years (Figure 3), although the analysis with the Chi-square test did not demonstrate this reduction (Table 5). In 2017, only 13 of the 97 papers presented were published (13.4%). During this period, the dissemination and awards of the event increased. Thus, one of the justifications for this trend is the increase in Brazilian participation in international congresses, such as the San Antonio Breast Cancer Symposium and American Society of Clinical Oncology (ASCO) Annual Meeting, which give researchers greater prestige and visibility [1, 6]. Given this information, researchers should be encouraged to present their abstracts at Brazilian events and, later, receive institutional support to publish them.

The publication rate found for papers presented at the BBCS is in agreement with bibliometric studies conducted with other medical specialities in Brazil [1, 8] but lower than that found for renowned international congresses [2, 16, 17]. The differences between this event and others held in Brazil for which publication rates are below 10% [9, 18] may be due to the quality control applied by the respective scientific committees reviewing and selecting the papers submitted and to the methodological characteristics of the papers accepted for presentation at the congress [3, 19]. Nevertheless, the fact that the BBCS is an event that focuses on scientific research and that offers a range of incentives to speakers and considerable awards and prizes for the best papers presented may also have contributed to this better performance [3].

Only three of the factors capable of affecting publication rates were found to be significant. The publication rate was higher in the case of studies developed in public institutions, possibly due to the authors’ academic experience and the scientific incentives present in the majority of these institutions in Brazil [7, 12, 13]. Studies dealing with rehabilitation following breast cancer were also more likely to be published, reflecting not only the quality of these studies but also the encouragement provided for multidisciplinary participation in the BBCS [3]. Finally, the publication rate was higher for papers given as oral or commented poster presentations, reflecting the effectiveness of the event’s organising committee in selecting the studies with greatest scientific impact [16, 19]. In a study conducted in Germany, three different factors were found to affect publication: positive findings, the editorial activity of the supervisor of the study and the quality of the statistical analysis [20]. Those factors were not evaluated in the present study because the information was not available; however, they should be the focus of future studies in this same line of research.

To the best of the authors’ knowledge, this is the first bibliometric survey of events related to breast cancer in Brazil. However, this study also highlights other relevant aspects. Despite the diversity of authors and institutions of origin, there was a predominance of studies originating in Goiás. This can probably be explained by the fact that the event takes place in that state and emphasises the importance of this symposium for the scientific development of the region [3]. There was also a predominance of papers on studies conducted in public hospitals and universities, reflecting the importance of these institutions in scientific production in Brazil [13]. Of the studies that went on to be published, it should be noted that *Mastology*, the journal of the Brazilian Breast Society, was the publication that most benefitted from papers presented at the BBCS. This corroborates the institutional support for the dissemination of Brazil’s scientific production and confirms the importance of the medical societies in the development and dissemination of academic knowledge [21].

Some limitations to the present study that must be mentioned include those inherent to any study conducted using secondary data sources such as the retrospective design and the limited access to some variables that could have added further information to the discussion. The cut-off point of 3 years after the last event included in the study minimises the risk of a temporal bias that could have existed in relation to the publication rate, although other studies have used 4–5 years [14, 22]. Thus, the publication rates for the 2016 and 2017 events may still increase slightly, in the coming years. On the other hand, the standardisation of the methodology and the rigour applied in the search for the papers increases the robustness of the data found in this study, which constitutes the first bibliometric survey in the field of breast cancer in Brazil.

## Conclusion

The publication rate of papers presented at the BBCS is low and remains consistent over the study period despite academic incentives and substantial awards. Studies conducted in public educational institutions, presented in the form of an oral presentation and addressing rehabilitation after breast cancer were associated with the highest publication rate.

## Conflicts of interest

None declared.

## Funding

This research did not receive any specific grant from funding agencies in the public, commercial or not-for-profit sectors.

## Authors’ contributions

Nascimento S contributed to the conception of the study, the acquisition, analysis and interpretation of the data, and manuscript writing. Rahal RMS, Soares LR, Pimentel HJS, Kamimura TO and Freitas-Junior R contributed equally to the conception and design of the study and to revising the manuscript critically for important intellectual content. All authors approved the final version of the manuscript and take public responsibility for the appropriate parts of the content.

## Figures and Tables

**Table 1.** Characteristics of the authors’ institution and of the presentation as a function of whether the paper was published, 2012–2017.

	Paper published? *n* (%)		Total	*p*-value^a^
	No 431 (79.4)	Yes 112 (20.6)		
**Type of institution**				
Public-private partnership	38 (8.8)	5 (4.5)	43 (7.9)	0.01
Private	103 (23.9)^b^	11 (9.8)	114 (21.0)	
Public	290 (67.3)	96 (85.7)^b^	386 (71.1)	
**Location of institution**				
State of Goiás	209 (48.5)	48 (42.9)	257 (47.3)	0.28
Another state	222 (51.5)	64 (57.1)	286 (52.7)	
**Type of presentation**				
Oral	54 (12.5)	33 (29.5)^b^	87 (16.0)	<0.001
Commented poster	44 (10.2)	21 (18.8)^b^	65 (12.0)	
Poster	333 (77.3)^b^	58 (51.8)	391 (72.0)	
**Subject matter**				
Epidemiology	90 (20.9)	16 (14.3)	106 (19.5)	0.04
Histology	55 (12.8)	16 (14.3)	71 (13.1)	
Basic science	46 (10.7)	16 (14.3)	62 (11.4)	
Surgery	46 (10.7)	14 (12.5)	60 (11.0)	
Rehabilitation	39 (9.0)	19 (17.0)^b^	58 (10.7)	
Radiology	25 (5.8)	10 (8.9)	35 (6.4)	
Systemic treatment	23 (5.3)	5 (4.5)	28 (5.2)	
Radiotherapy	12 (2.8)	3 (2.7)	15 (2.8)	
Others	95 (22.0)^b^	13 (11.6)	108 (19.9)	

a Pearson’s chi-square test

b Post hoc test

*n*, Absolute frequency; %, Relative frequency

**Table 2.** Comparison between the type of institution and characteristics of the publication.

	Type of institution, *n* (%)			Total	*p*-value^a^
	Private	Public	Public-private partnership		
**Language of publication**					
Spanish	0 (0.0)	0 (0.0)	1 (20.0)	1 (0.9)	0.10
English	6 (54.5)	68 (70.8)	1 (20.0)	75 (67.0)	
Portuguese	5 (45.5)	28 (29.2)	3 (60.0)	36 (32.1)	
**Agreement (congress/paper)**					
In agreement	6 (54.5)	37 (38.5)	2 (40.0)	45 (40.2)	0.89
Major disagreement	2 (18.2)	20 (20.8)	1 (20.0)	23 (20.5)	
Slight disagreement	3 (27.3)	39 (40.6)	2 (40.0)	44 (39.3)	
**Degree of recommendation**					
A	1 (9.1)	8 (8.3)	0 (0.0)	9 (8.0)	0.75
B	8 (72.7)	70 (72.9)	3 (60.0)	81 (72.3)	
C	1 (9.1)	4 (4.2)	1 (20.0)	6 (5.4)	
D	1 (9.1)	14 (14.6)	1 (20.0)	16 (14.3)	
**Time to publication**					
Publication preceded congress	1 (9.1)	7 (7.3)	1 (20.0)	9 (8.0)	0.42
Same year	4 (36.4)	13 (13.5)	1 (20.0)	18 (16.1)	
1–2 years	5 (45.5)	47 (49.0)	2 (40.0)	54 (48.2)	
≥3 years	1 (9.1)	29 (30.2)	1 (20.0)	31 (27.7)	

a Chi-square test

*n*, Absolute frequency, %, Relative frequency

**Table 3.** Comparison between time to publication and characteristics of the journal and the article.

	Time to publication, *n* (%)				*p*-value^a^
	Publication preceded congress	Same year	1–2 years	>3 years	
**Language of publication**					
Spanish	0 (0.0)	0 (0.0)	0 (0.0)	1 (3.2)	0.30
English	4 (44.4)	10 (55.6)	40 (74.1)	21 (67.7)	
Portuguese	5 (55.6)	8 (44.4)	14 (25.9)	9 (29.0)	
**Agreement (congress/paper)**					
In agreement	3 (33.3)	7 (38.9)	25 (46.3)	10 (32.3)	0.57
Major disagreement	3 (33.3)	2 (11.1)	9 (16.7)	9 (29.0)	
Slight disagreement	3 (33.3)	9 (50.0)	20 (37.0)	12 (38.7)	
**Degree of scientific evidence**					
A	1 (11.1)	2 (11.1)	3 (5.6)	3 (9.7)	0.67
B	6 (66.7)	14 (77.8)	39 (72.2)	22 (71.0)	
C	0 (0.0)	1 (5.6)	5 (9.3)	0 (0.0)	
D	2 (22.2)	1 (5.6)	7 (13.0)	6 (19.4)	

a Chi-square test

*n*, Absolute frequency; %, Relative frequency

**Table 4.** Names of the journals in which the papers were published as a function of the proportion of publications.

Names of the journals	*n* (%)
Revista Brasileira de Mastologia/Mastology	27 (24.1)
The Breast Journal	5 (4.4)
*RBGO Gynecology and Obstetrics*	3 (2.7)
BMC Public Health	3 (2.7)
Breast Cancer Research and Treatment	3 (2.7)
Supportive Care in Cancer	3 (2.7)
European Journal of Obstetrics, Gynecology, and Reproductive Biology	2 (1.8)
The Breast (Edinburgh)	2 (1.8)
Revista do Colégio Brasileiro de Cirurgiões	2 (1.8)
Asian Pacific Journal of Cancer Prevention	2 (1.8)
Journal of Radiological Protection	2 (1.8)
Annals of Surgical Oncology	2 (1.8)
Clinical Breast Cancer	2 (1.8)
Molecular Medicine Reports	2 (1.8)
Others^a^	52 (46.4)

Note that the ‘*Revista Brasileira de Mastologia’* changed its name to ‘*Mastology’* in April 2017, within the study period

a Others: Journals for which the publication rate was < 1%

**Table 5.** Characteristics of the institution according to year of presentation (2012 and 2017).

	Year *n* (%)						*p*-value^a^
	2012	2013	2014	2015	2016	2017	
**Type of institution**							
Public-private partnership	12 (22.6)^b^	6 (7.7)	9 (9.0)	4 (4.1)	4 (3.4)	8 (8.2)	<0.001
Private	3 (5.7)	25 (32.1)^b^	17 (17.0)	29 (29.9)^b^	27 (22.9)	13 (13.4)	
Public	38 (71.7)	47 (60.3)	74 (74.0)	64 (66.0)	87 (73.7)	76 (78.4)	
**Type of presentation**							
Oral	13 (24.5)	15 (19.2)	15 (15.0)	15 (15.5)	14 (11.9)	15 (15.5)	0.01
Poster	40 (75.5)	63 (80.8)	65 (65.0)	67 (69.1)	89 (75.4)	67 (69.1)	
Commented poster	0 (0.0)	0 (0.0)	20 (20.0)^b^	15 (15.5)^b^	15 (12.7)	15 (15.5)^b^	
**Location of institution**							
State of Goiás	26 (49.1)	50 (64.1)^b^	35 (35.0)	48 (49.5)	43 (36.4)	55 (56.7)^b^	0.001
Another state	27 (50.9)	28 (35.9)	65 (65.0)	49 (50.5)	75 (63.6)	42 (43.3)	
**Subject matter**							
Basic science	2 (3.8)	4 (5.1)	12 (12.0)	11 (11.3)	17 (14.4)	16 (16.5)	0.06
Surgery	10 (18.9)	10 (12.8)	10 (10.0)	9 (9.3)	10 (8.5)	11 (11.3)	
Epidemiology	8 (15.1)	21 (26.9)	14 (14.0)	23 (23.7)	26 (22.0)	14 (14.4)	
Histology	8 (15.1)	12 (15.4)	13 (13.0)	13 (13.4)	7 (5.9)	18 (18.6)	
Radiology	3 (5.7)	8 (10.3)	5 (5.0)	6 (6.2)	8 (6.8)	5 (5.2)	
Radiotherapy	2 (3.8)	1 (1.3)	2 (2.0)	7 (7.2)	1 (0.8)	2 (2.1)	
Rehabilitation	9 (17.0)	10 (12.8)	16 (16.0)	3 (3.1)	16 (13.6)	4 (4.1)	
Systemic treatment	2 (3.8)	2 (2.6)	4 (4.0)	8 (8.2)	7 (5.9)	5 (5.2)	
Others	9 (17.0)	10 (12.8)	24 (24.0)	17 (17.5)	26 (22.0)	22 (22.7)	
**Paper published?**							
No	36 (67.9)	57 (73.1)	72 (72.0)	83 (85.6)	99 (83.9)	84 (86.6)	0.07
Yes	17 (32.1)	21 (26.9)	28 (28.0)	14 (14.4)	19 (16.1)	13 (13.4)	

a Chi-square test

b Post hoc test

*n*, Absolute frequency; %, Relative frequency

## References

[1] Pinheiro CMA, Masson ALS, Faingezicht AM (2009). Estudos Brasileiros Apresentados nos Encontros Anuais da ASCO entre 2001 e 2007: Aumento de Produção, com Baixa Taxa de Publicação. Rev Bras Cancerol.

[2] Arap MA, Reis RB, Torricelli FC (2014). Brazilian abstracts presented at the American Urological Association annual meetings: contribution, publication rates, and comparison with oncology abstracts. Int Braz J Urol.

[3] Rahal RMS, Nascimento S, Soares LR (2020). Publication rate of abstracts on breast cancer presented at different scientific events in Brazil. Mastology.

[4] Simon SD, Bines J, Werutsky G (2019). Characteristics and prognosis of stage I-III breast cancer subtypes in Brazil: the AMAZONA retrospective cohort study. Breast.

[5] Freitas-Junior R, Nunes RD, Martins E (2017). Prognostic factors and overall survival of breast cancer in the city of Goiania, Brazil: a population-based study. Rev Col Bras Cir.

[6] Freitas PF, Freitas-Junior RF, Soares LR (2014). Série temporal da apresentação de trabalhos brasileiros no San Antonio Breast Cancer Symposium. Rev Bras Mastologia.

[7] Younes RN, Deheinzelin D, Birolini D (2005). Graduate education at the faculty of medicine of the University of Sao Paulo: quo vadis?. Clinics.

[8] Denadai R, Pinho AS, Júnior SH (2017). Conversion of plastic surgery meeting abstract presentations to full manuscripts: a Brazilian perspective. Rev Col Bras Cir.

[9] Bonetti W, Holmo NF, Corregliano GT (2008). Publicações indexadas geradas a partir de resumos de congressos de angiologia e cirurgia vascular no Brasil. J Vasc Bras.

[10] MacDonald PL, Gardner RC (2000). Type I error rate comparisons of post hoc procedures for I j Chi-Square tables. Educ Psychol Meas.

[11] Santos EF, Pereira MG (2007). Qualidade dos resumos estruturados apresentados em congresso médico. Rev Assoc Med Bras.

[12] Zorzetto R, Razzouk D, Dubugras MT (2006). The scientific production in health and biological sciences of the top 20 Brazilian universities. Braz J Med Biol Res.

[13] Cardoso SC, Gattás GJF (2009). The scientific production of full professors of the Faculdade de Medicina da Universidade de São Paulo: a view of the period of 2001–2006. Clinics.

[14] Denadai R, Araujo GH, Pinho AS (2016). Discrepancies between plastic surgery meeting abstracts and subsequent full-length manuscript publications. Aesthetic Plast Surg.

[15] Sarkar B, Wang YX, Cai J (2020). The open access financial model hinders the growth of medical physics research in low- and middle-income countries. Med Phys.

[16] Ohtori S, Orita S, Eguchi Y (2018). Oral presentations have a significantly higher publication rate, but not impact factors, than poster presentations at the International Society for study of lumbar spine meeting: review of 1126 abstracts from 2010 to 2012 meetings. Spine (Phila Pa 1976).

[17] Meral UM, Alakus U, Urkan M (2016). Publication rate of abstracts presented at the annual congress of the European Society for Surgical Research during 2008–2011. Eur Surg Res.

[18] de Andrade VA, Carpini S, Schwingel R (2011). Publication of papers presented in a Brazilian Trauma Congress. Rev Col Bras Cir.

[19] Vieira RAC, Bonetti TCS, Marques MMC (2020). Criteria for evaluating studies at scientific medical events. Mastology.

[20] Vogel U, Windeler J (2000). Factors modifying frequency of publications of clinical research results exemplified by medical dissertations. Dtsch Med Wochenschr.

[21] Urban C (2019). Mastology in the open entrance of the closed palace of the king. Mastology.

[22] Hackett PJ, Guirguis M, Sakai N (2014). Fate of abstracts presented at the 2004–2008 International Liver Transplantation Society meetings. Liver Transpl.

